# Crystal structures of two platinum(II) complexes containing ethyl eugenoxyacetate and 2-amino­pyridine

**DOI:** 10.1107/S2056989017004285

**Published:** 2017-03-24

**Authors:** Hai Le Thi Hong, Thao Nguyen Thu, Hien Nguyen, Luc Van Meervelt

**Affiliations:** a Department of Chemistry, Hanoi National University of Education, 136 Xuan Thuy, Cau Giay, Hanoi, Vietnam; b Department of Chemistry, KU Leuven, Biomolecular Architecture, Celestijnenlaan 200F, Leuven (Heverlee), B-3001, Belgium

**Keywords:** crystal structure, platinum(II) complexes, 2-amino­pyridine, eugenol

## Abstract

In the title complexes, *trans*-(2-amino­pyridine-κ*N*)di­chlorido­{4-eth­oxy­carbonyl­meth­oxy-3-meth­oxy-1-[(2,3-η)-prop-2-en-1-yl]benzene}­platinum(II), [PtCl_2_(C_5_H_6_N_2_)(C_14_H_18_O_4_)], (I), and (2-amino­pyridine-κ*N*)chlorido{5-eth­oxy­car­bon­yl­meth­oxy-4-meth­oxy-1-[(2,3-η)-prop-2-en-1-yl]phenyl-κ*C* ^1^}­platinum(II), [Pt(C_14_H_17_O_4_)Cl(C_5_H_6_N_2_)], (II), the central Pt^II^ metal atom displays a distorted square-planar coordination, with the Pt^II^ atom coordinated by the pyridine N atom, the C=C double bond of the eugenol ligand and two Cl atoms for (I) or one Cl atom and a C atom of the phenyl ring for (II). The allyl fragment in (I) is disordered, with population parameters 0.614 (14) and 0.386 (14) for the two positions of the central C atom. The least-squares planes through the two aromatic ring systems make a dihedral angle of 51.10 (13)° for (I) and 78.5 (2)° for (II). Intra­molecular N—H⋯O and N—H⋯π inter­actions occur in (I). In (I), inversion dimers formed by C—H⋯Cl inter­actions are further linked into chains parallel to the *b* axis by C—H⋯O hydrogen bonds. Both aromatic rings are involved in π–π inter­actions, with centroid-to-centroid distances of 3.508 (3) and 3.791 (3) Å. In (II), inversion dimers form chains parallel to the *b* axis by C—H⋯O inter­actions.

## Abstract 2

The synthesis and crystal structures of two platinum(II) complexes containing one or two Cl atoms, an eugenol derivative and 2-amino­pyridine as ligand are described. The central Pt^II^ atom displays a distorted square-planar coordination.

## Chemical context   

Since the discovery of the anti­cancer activity and subsequent clinical success of cisplatin {*cis*-[PtCl_2_(NH_3_)_2_]}, platinum-based compounds have been widely synthesized and studied as potential chemotherapeutic agents (Wong & Giandomenico, 1999 ▸). Despite the great success in treating certain kinds of cancer, there are several side effects, and both intrinsic and acquired resistance limit the organotropic profile of the drug (Chabner & Roberts, 2005 ▸; Kelland, 2007 ▸; Wilson & Lippard, 2014 ▸). Hence, there is continuing inter­est in the development of new platinum complexes that have high activities but low toxicity (Johnstone *et al.*, 2014 ▸).

Several natural aryl­olefins, such as safrole (in sassafras oil), eugenol (in clove oil) and anethole (in anise and fennel oil), and their derivatives have been used as important inter­mediate materials to synthesize many compounds that have various applications in the flavouring, food and pharmaceutical industries (Jadhav *et al.*, 2004 ▸). Recently, a number of Pt^II^ complexes containing natural aryl­olefins as ligands, *i.e.* safrole or derivatives of eugenol such as methyl­eugenol and alkyl­eugenoxyacetate, have been prepared (Da *et al.*, 2010 ▸, 2012 ▸; Da, Chi *et al.*, 2015 ▸; Da, Hai *et al.*, 2015 ▸; Nguyen Thi Thanh *et al.*, 2016 ▸; Le Thi Hong *et al.*, 2016 ▸). The insertion of these natural aryl­olefins into the coordination with Pt^II^ and their transformations formed complexes with novel structures and high applicability. In particular, many of these organoplatinum(II) complexes exhibit significant inhibitory activities against human cancer cells (Da *et al.*, 2012 ▸; Da, Chi *et al.*, 2015 ▸; Da, Hai *et al.*, 2015 ▸).
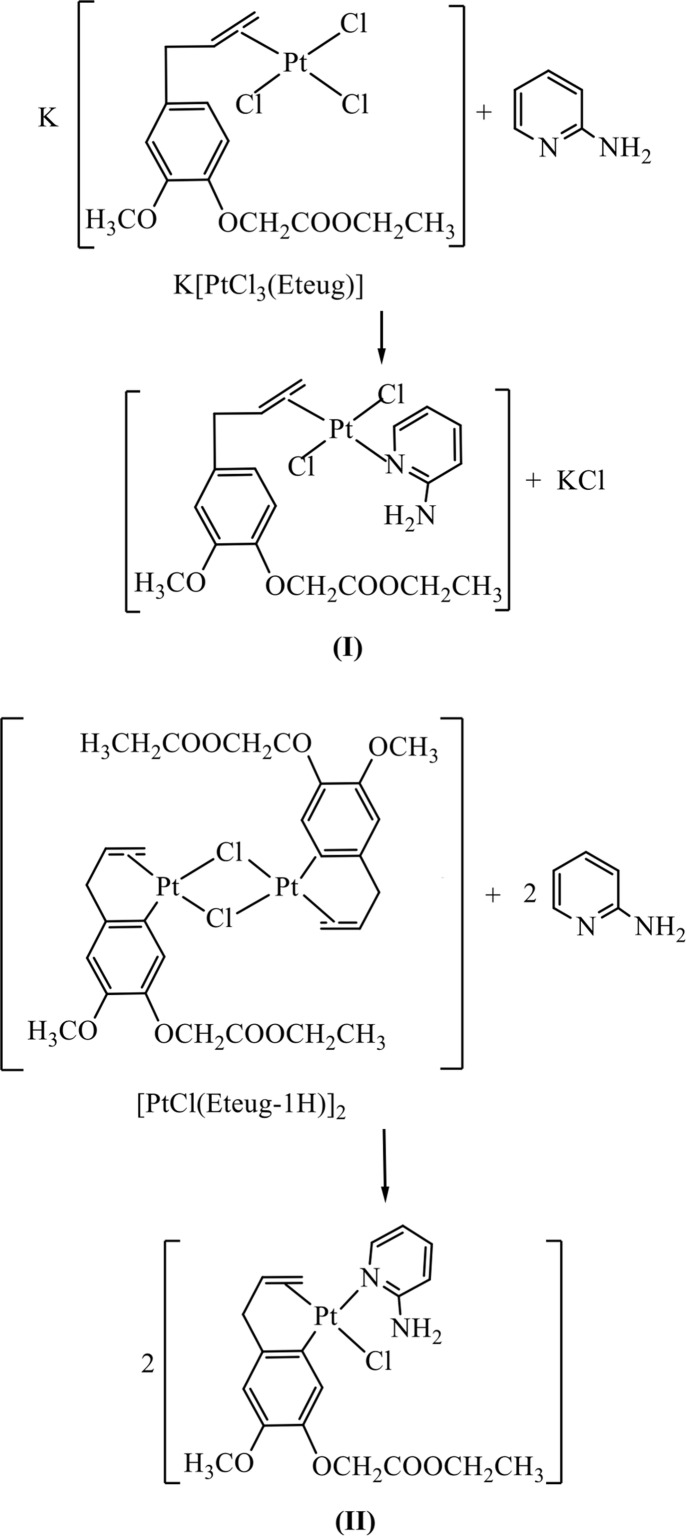


Herein, we report the syntheses and crystal structure determinations of organoplatinum(II) complexes formed by the complexation of 2-amino­pyridine as ligand with the mononuclear platinum(II) complex K[PtCl_3_(Eteug)] and the binuclear platinum(II) complex [Pt_2_(Eteug-1H)_2_Cl_2_] (Eteug is ethyl­eugenoxyl­acetate).

## Structural commentary   

**Figure 1:**
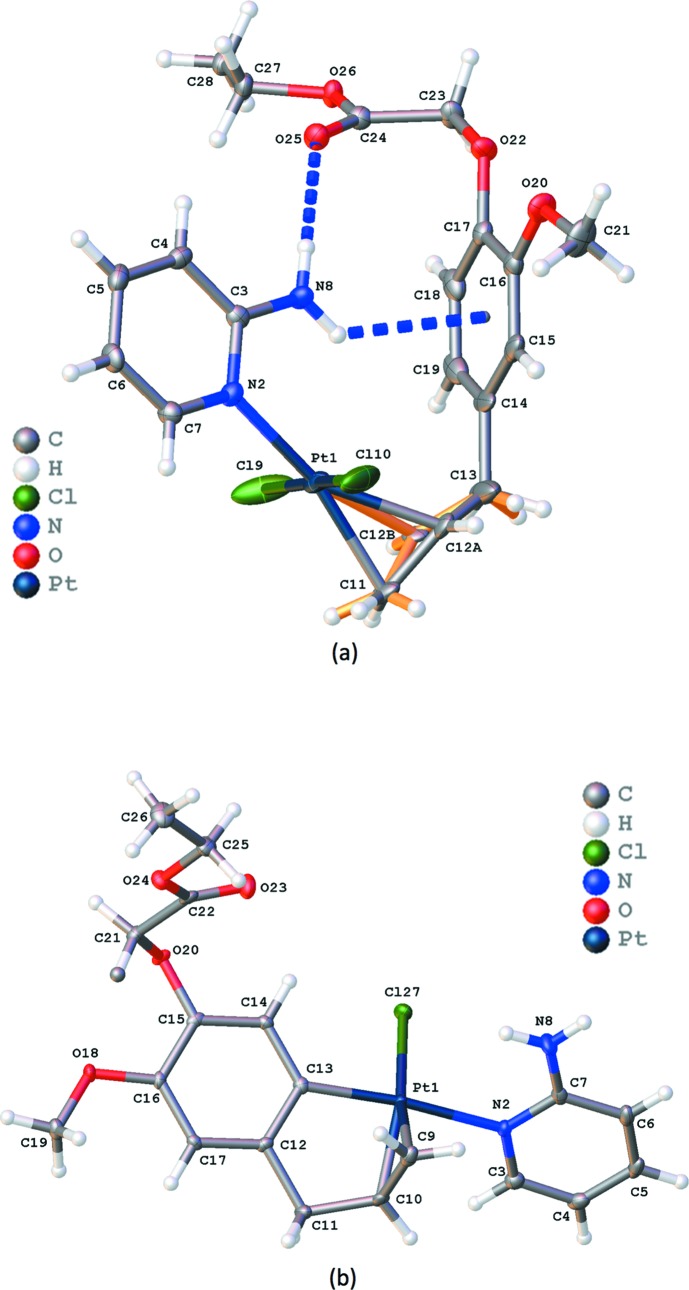
Views of the asymmetric units in (*a*) (I) and (*b*) (II), showing the atom-labelling schemes. Displacement ellipsoids are drawn at the 50% probability level. Orange bonds in (*a*) show the allyl fragment with a population parameter of 0.386 (14). Blue dotted lines indicate intra­molecular inter­actions in (*a*).

**Figure 2:**
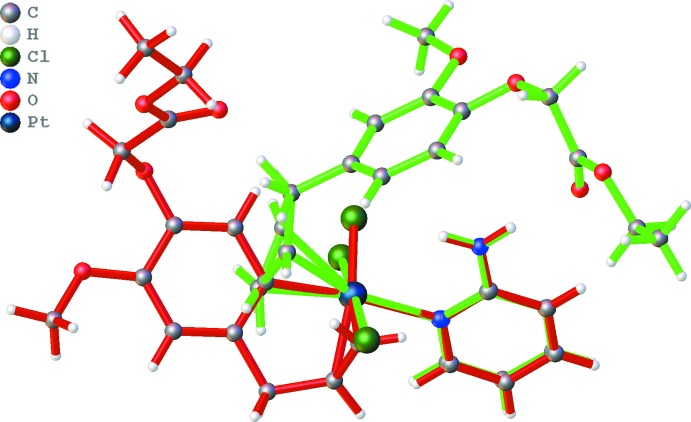
Overlay of the Pt–2-amino­pyridine fragment present in (I) (green bonds) and (II) (red bonds).

In both title complexes, the central Pt^II^ metal atom displays a distorted square-planar coordination (Fig. 1 ▸). In addition to the two Cl atoms in dichloride complex (I), the pyridine N atom and the C=C double bond of the eugenol ligand coordinate to the central Pt^II^ atom. In monochloride complex (II), one Cl atom is replaced by a C atom of the eugenol phenyl group. An overlay of the Pt–2-amino fragment present in both structures clearly shows the differences in coordination (Fig. 2 ▸). Where in (I) the Cl atoms are *trans* with respect to each other, this is the case for the two aromatic rings in (II). One Cl and the C=C coordinations in (I) are replaced by, respectively, C=C and a phenyl C atom in (II). In both cases, the 2-amino­pyridine ligand only inter­acts *via* the ring N atom. In (I), the CH_2_—CH=CH_2_ fragment is disordered, with population parameters of 0.614 (14) and 0.386 (14) for the two positions of the central C atom. The dihedral angles between the planes through the two aromatic rings are 78.5 (2) and 51.10 (13)° for (I) and (II), respectively. In (I), the H atoms of the amino group are involved in a weak intra­molecular N—H⋯O inter­action (N8—H8*B*⋯O25, Table 1 ▸) and an N—H⋯π inter­action (N8—H8*A*⋯*Cg*1, Table 1 ▸; *Cg*1 is the centroid of the C14–C19 ring). Similar inter­actions are not possible in (II) due to the different orientation of the ligands.

## Supra­molecular features   

The complexes crystallize in different space groups, *viz. P* 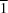 for di­chloride complex (I) and *P*2_1_/*c* for monochloride complex (II).

**Figure 3:**
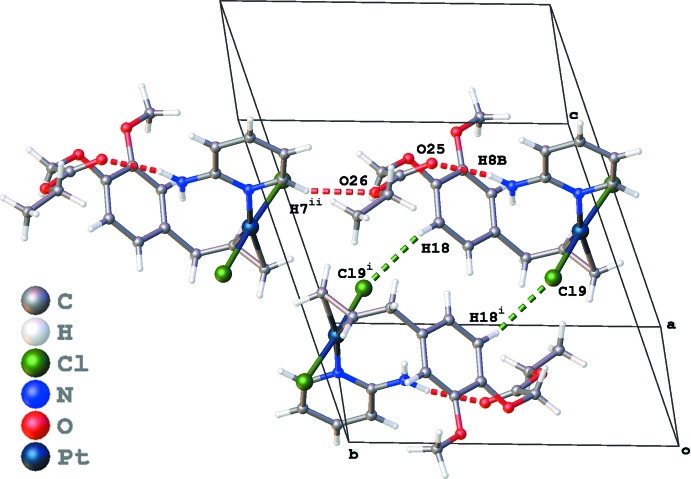
Packing diagram of (I), showing the C—H⋯O (red dotted lines) and C—H⋯Cl inter­actions (green dotted lines). [Symmetry codes: (i) −*x*, −*y* + 1, −*z* + 1; (ii) *x*, *y* − 1, *z*.]

**Figure 4:**
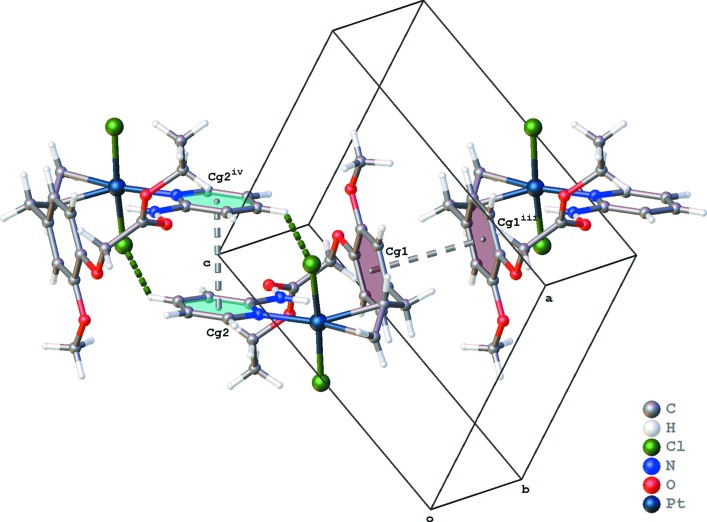
Partial packing diagram of (I), showing π–π stacking (gray dotted lines). [*Cg*1 and *Cg*2 are the centroids of the C14–C19 and N2/C3–C7 rings; symmetry codes: (iii) −*x* + 1, −*y* + 1, −*z* + 1; (iv) −*x*, −*y*, −*z* + 2.]

The crystal packing of (I) is dominated by hydrogen bonding and π–π inter­actions. Inversion dimers formed by C18—H18⋯Cl9^i^ hydrogen bonds are further linked into chains parallel to the *b* axis by C7—H7⋯O26^ii^ hydrogen bonds [Table 1 ▸ and Fig. 3 ▸; symmetry codes: (i) *x*, *y* − 1, *z*; (ii) −*x*, −*y* + 1, −*z* + 1]. Both aromatic rings show π–π stacking, with *Cg*1⋯*Cg*1^iii^ = 3.791 (3) Å for the phenyl ring and *Cg*2⋯*Cg*2^iv^ = 3.508 (3) Å for the pyridine ring [*Cg*1 and *Cg*2 are the centroids of the C14–C19 and N2/C3–C7 rings; symmetry codes: (iii) −*x* + 1, −*y* + 1, −*z* + 1; (iv) −*x*, −*y*, −*z* + 2; Fig. 4 ▸].

**Figure 5:**
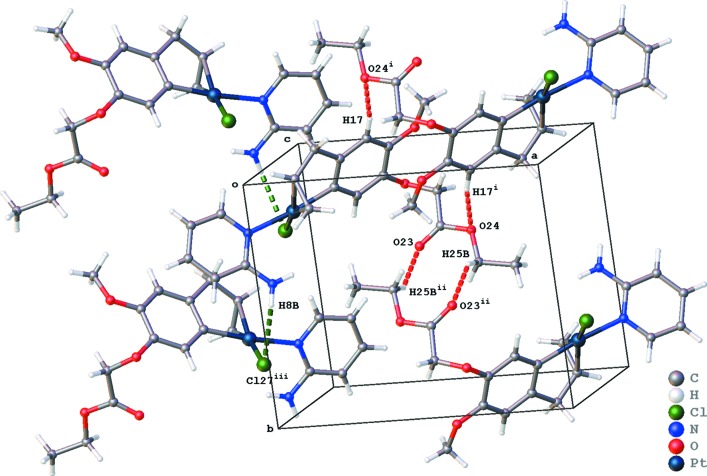
Partial packing diagram of (II), showing the C—H⋯O (red dotted lines), N—H⋯Cl (green dotted lines) and C—H⋯π (gray dotted lines) inter­actions. [Symmetry codes: (i) −*x* + 1, −*y*, −*z* + 1; (ii) −*x* + 1, −*y* + 1, −*z* + 1; (iii) −*x*, *y* + 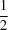, −*z* + 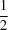.]

The crystal packing of (II) is built up by C—H⋯O, N—H⋯Cl and C—H⋯π inter­actions (Table 2 ▸ and Fig. 5 ▸). Two types of inversion dimers are created by C—H⋯O inter­actions enclosing 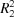
(10) and 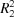
(16) ring motifs, and resulting in the formation of chains parallel to the *b* axis. No π–π inter­actions are observed in the packing of (II).

## Database survey   

The Pt—N distances of 2.066 (3) Å in (I) and 2.143 (2) Å in (II) agree well with the average Pt—N distance of 2.06 (7) Å for Pt–pyridine fragments present in the Cambridge Structural Database (CSD, Version 5.38, last update February 2017; Groom *et al.*, 2016 ▸).

The CSD contains 34 Pt complexes with Pt coordinated by Cl, pyridine and C=C, with 28 complexes having an additional Cl atom as the fourth ligand (27 *trans* and one *cis* coordination), three a C atom and another three an N atom.

The synthesis of (II), starting from the dinuclear complex [Pt_2_Cl_2_(Eteug-1H)_2_], can be rationalized by the replacement of the Cl atom in the *trans* position with respect to the C=C bond. Verification of the Pt—Cl distances in the dinuclear complex di-μ-chlorido-bis­[(η^2^-2-allyl-4-meth­oxy-5-{[(propan-2-yl­oxy)carbon­yl]meth­oxy}phenyl-κ*C* ^1^)platinum(II)], [Pt_2_(IsoPreug-1H)_2_Cl_2_] (IsoPreug-1H is isopropyleugenoxylacetate; CSD refcode EWAVIJ; Nguyen Thi Thanh *et al.*, 2016 ▸) indicates that the longest Pt—Cl bond [2.4773 (7) *versus* 2.3527 (7) Å] is cleaved, leading to a *cis* position of 2-amino­pyridine with respect to the C=C bond.

## Synthesis and crystallization   

### Synthesis of K[PtCl_3_(Eteug)] and [Pt_2_Cl_2_(Eteug-1H)_2_]   

The mononuclear complex K[PtCl_3_(Eteug)] and the dinuclear chelate ring complex [Pt_2_Cl_2_(Eteug-1H)_2_] were synthesized following the protocol of Da and co-workers (Da *et al.*, 2012 ▸; Da, Chi *et al.*, 2015 ▸; Da, Hai *et al.*, 2015 ▸).

### Synthesis of *trans*-[PtCl_2_(Eteug)(C_5_H_6_N_2_)], (I)   

While stirring, a solution of 2-amino­pyridine (0.22 mmol) in acetone (2 ml) was added slowly to a solution of K[PtCl_3_(Eteug)] (0.2 mmol) in acetone (15 ml). After 2 h, a white precipitate of KCl was separated out. After stirring for 3 h at room temperature, ethanol (2 ml) was added to the obtained solution. Slow evaporation of the solvent at room temperature afforded the desired product as bright orange–yellow crystals. The yield was 80%. The product is soluble in acetone and chloro­form, but only slightly soluble in ethanol and insoluble in water. Single crystals suitable for X-ray diffraction were obtained from an acetone/ethanol (3:1 *v*/*v*) solution *via* slow evaporation of the solvents at 277–278 K.

### Data for trans-[PtCl_2_(Eteug)(C_5_H_6_N_2_)], (I)   

IR (Impack-410 Nicolet spectrometer, KBr, cm^−1^): 3454, 3341 (ν_NH_); 3060, 2930 (ν_CH_); 1739 (ν_C=O_); 1598, 1512 (aromatic, ν_C=C_, ν_C= N_).

^1^H NMR (δ p.p.m.; Bruker AVANCE 500 MHz, CDCl_3_): 7.15 (1H, *d*, ^4^ *J* = 1.5 Hz, Ar), 7.00 (1H, *t*, ^3^ *J* = 8.0 Hz, ^4^ *J* = 1.5 Hz, Ar), 6.77 (1H, *d*, ^3^ *J* = 8 Hz, Ar), 4.82 (1H, *d*, ^2^ *J* = 17 Hz, *OCH_2_*), 4.77 (1H, *d*, ^2^ *J* = 16.5 Hz, OCH_2_) , 3.91 (3H, *s, OCH_3_*), 4.28 (2H, *q*, ^3^ *J* = 7 Hz, -CH_2_CH_3_), 1.33 (3H, *t*, ^3^ *J* = 7 Hz, CH_2_CH_3_), 3.26 (1H, *dd*, ^2^ *J* = 15 Hz, ^3^ *J* = 7.5 Hz, CH_2_CH), 3.39 (1H, *dd*, ^2^ *J* = 15 Hz, ^3^ *J* = 7.5 Hz, CH_2_CH), 5.99 (1H, *m*, ^2^ *J*_PtH_ = 70 Hz, CH=CH_2_), 4.69 (1H, *d*, ^3^ *J* = 8, ^2^ *J*_PtH_ = 70 Hz, *cis*-alkene), 4.78 (1H, *ov*, *trans*-alkene), 6.47 (1H, *d*, ^3^ *J* = 8.5 Hz, Ar of 2-amino­pyridine), 6.6 (1H, *t*, ^3^ *J* = 6 Hz, Ar), 7.35 (1H, *m*, ^3^ *J* = 7.5 Hz, ^4^ *J* = 1.5 Hz, Ar), 7.86 (1H, *d*, ^3^ *J* = 6 Hz, Ar), 5.21 (*ov*, NH_2_).

### Synthesis of [Pt(Eteug-1H)Cl(C_5_H_6_N_2_)], (II)   

A solution of 2-amino­pyridine (0.22 mmol) in acetone (2 ml) was added slowly to a mixture of [Pt_2_(Eteug-1H)_2_Cl_2_] (0.1 mmol) and acetone/ethanol (6 ml, 1:2 *v*/*v*). After stirring for 2 h at room temperature, a yellow solution was obtained. A white precipitate was formed by slow evaporation of the solvent at 277–278 K. The precipitate was collected by filtration and washed with ethanol. The product is soluble in acetone and chloro­form, but only slightly soluble in ethanol and insoluble in water. The yield was 75%. Single crystals suitable for X-ray diffraction were obtained from a acetone/ethanol (1:1 *v*/*v*) solution *via* slow evaporation of the solvents at 277–278 K.

### Data for [Pt(Eteug-1H)Cl(C_5_H_6_N_2_)], (II)   

IR (Impack-410 Nicolet spectrometer, KBr, cm^−1^): 3446, 3332 (ν_NH_); 3070, 2941, 2849 (ν_CH_); 1756 (ν_C=O_); 1566 (aromatic, ν_C=C_, ν_C=N_).

^1^H NMR (δ p.p.m.; Bruker AVANCE 500 MHz, CD_3_COCD_3_): 6.66 (1H, *s*, Ar), 7.04 (1H, *s*, ^3^ *J*_PtH_ = 40 Hz, Ar), 4.59 (1H, *d*, ^2^ *J* = 16 Hz, *2H, OCH_2_*), 4.55 (1H, *d*, ^2^ *J* = 16 Hz, *OCH_2_*), 3.73 (3H, *s*, ^3^ *J* = 7 Hz, OCH_3_), 4.21 (2H, *m*, ^3^ *J* = 7 Hz, CH_2_CH_3_), 1.28 (3H, *t*, ^3^ *J* = 7.0 Hz, CH_2_CH_3_), 2.65 (1H, *d*, ^2^ *J* = 16.5; ^3^ *J*_PtH_ = 100 Hz, CH_2_CH), 3.78 (1H, *ov*, CH_2_CH), 4.74 (1H, *m*, ^2^ *J*_PtH_ = 75 Hz, CH=CH_2_), 3.72 (1H, *ov*, *cis*-alkene), 3.82 (1H, *d*, ^3^ *J* = 13.5 Hz, *trans*-alkene), 6.85 (1H, *d*, ^3^ *J* = 8.5 Hz, Ar of 2-amino­pyridine), 6.72 (1H, *m*, Ar), 7.56 (1H, *m*, Ar), 8.07 (1H, *d*, ^3^ *J* = 6 Hz, Ar), 6.43 (*ov*, NH_2_).

## Refinement   

Crystal data, data collection and structure refinement details are summarized in Table 3 ▸. All H atoms were placed in idealized positions and refined in the riding mode, with *U*_iso_(H) values assigned as 1.2*U*_eq_ of the parent atoms (1.5 times for methyl groups), and with C—H distances of 0.95 (aromatic and =CH_2_), 0.98 (CH_3_), 0.99 (CH_2_) and 1.00 Å (CH), and N—H distances of 0.88 Å (NH_2_).

In (I), the central C atom in the CH_2_—CH=CH_2_ fragment is disordered over two positions [population parameters = 0.614 (14) and 0.386 (14)] and was refined with constraints for bond lengths and anisotropic displacement parameters present in this fragment.

## Supplementary Material

Crystal structure: contains datablock(s) I, II. DOI: 10.1107/S2056989017004285/rz5208sup1.cif

Structure factors: contains datablock(s) I. DOI: 10.1107/S2056989017004285/rz5208Isup2.hkl

Structure factors: contains datablock(s) II. DOI: 10.1107/S2056989017004285/rz5208IIsup3.hkl

CCDC references: 1538567, 1538566

Additional supporting information:  crystallographic information; 3D view; checkCIF report

## Figures and Tables

**Table 1:** Hydrogen-bond geometry (Å, °) for (I) *Cg*1 is the centroid of the C14–C19 ring.

*D*—H⋯*A*	*D*—H	H⋯*A*	*D*⋯*A*	*D*—H⋯*A*
N8—H8*B*⋯O25	0.88	2.19	3.054 (5)	167
C7—H7⋯O26^i^	0.95	2.49	3.258 (6)	138
C18—H18⋯Cl9^ii^	0.95	2.78	3.460 (5)	130
N8—H8*A*⋯*Cg*1	0.88	2.53	3.166 (4)	129

Symmetry codes: (i) 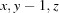; (ii) 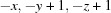.

**Table 2:** Hydrogen-bond geometry (Å, °) for (II) *Cg*2 is the centroid of the C12–C17 ring.

*D*—H⋯*A*	*D*—H	H⋯*A*	*D*⋯*A*	*D*—H⋯*A*
C17—H17⋯O24^i^	0.95	2.60	3.501 (3)	159
C25—H25*B*⋯O23^ii^	0.99	2.60	3.449 (3)	144
N8—H8*B*⋯Cl27^iii^	0.88	2.67	3.413 (2)	143
C19—H19*A*⋯*Cg*2^i^	0.98	2.63	3.476 (3)	145

Symmetry codes: (i) 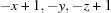; (ii) 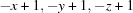; (iii) 
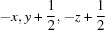
.

**Table 3:** Experimental details

	(I)	(II)
Crystal data		
Chemical formula	[PtCl_2_(C_5_H_6_N_2_)(C_14_H_18_O_4_)]	[Pt(C_14_H_17_O_4_)Cl(C_5_H_6_N_2_)]
*M*_r_	610.39	573.93
Crystal system, space group	Triclinic, *P* 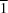	Monoclinic, *P*2_1_/*c*
Temperature (K)	100	100
*a*, *b*, *c* (Å)	8.3187 (3), 11.4119 (3), 12.1012 (4)	15.1198 (5), 10.0855 (2), 14.1855 (4)
α, β, γ (°)	70.437 (3), 73.688 (3), 87.038 (2)	90, 117.329 (4), 90
*V* (Å^3^)	1037.76 (6)	1921.72 (11)
*Z*	2	4
Radiation type	Mo *K*α	Mo *K*α
μ (mm^−1^)	7.05	7.47
Crystal size (mm)	0.30 × 0.30 × 0.15	0.30 × 0.30 × 0.20

Data collection		
Diffractometer	Agilent SuperNova diffractometer (single source at offset, Eos detector)	Agilent SuperNova diffractometer (single source at offset, Eos detector)
Absorption correction	Multi-scan (*CrysAlis PRO*; Rigaku Oxford Diffraction, 2015 ▸)	Multi-scan (*CrysAlis PRO*; Rigaku Oxford Diffraction, 2015 ▸)
*T*_min_, *T*_max_	0.406, 1.000	0.314, 1.000
No. of measured, independent and observed [*I* > 2σ(*I*)] reflections	21329, 4241, 4006	20268, 3928, 3715
*R*_int_	0.052	0.033
(sin θ/λ)_max_ (Å^−1^)	0.625	0.624

Refinement		
*R*[*F* ^2^ > 2σ(*F* ^2^)], *wR*(*F* ^2^), *S*	0.029, 0.065, 1.04	0.016, 0.038, 1.06
No. of reflections	4241	3928
No. of parameters	265	246
No. of restraints	26	0
H-atom treatment	H-atom parameters constrained	H-atom parameters constrained
Δρ_max_, Δρ_min_ (e Å^−3^)	1.41, −2.25	0.69, −1.04

Computer programs: *CrysAlis PRO* (Rigaku Oxford Diffraction, 2015 ▸), *SHELXS97* (Sheldrick, 2008 ▸), *SUPERFLIP* (Palatinus & Chapuis, 2007 ▸), *SHELXL2014* (Sheldrick, 2015 ▸) and *OLEX2* (Dolomanov *et al.*, 2009 ▸).

## supplementary crystallographic information

### (I) trans-(2-Aminopyridine-κN)dichlorido{4-ethoxycarbonylmethoxy-3-methoxy-1-[(2,3-η)-prop-2-en-1-yl]benzene}platinum(II) Crystal data

**Table:**

[PtCl_2_(C_5_H_6_N_2_)(C_14_H_18_O_4_)]	*Z* = 2
*M**_r_* = 610.39	*F*(000) = 592
Triclinic, *P*1	*D*_x_ = 1.953 Mg m^−^^3^
*a* = 8.3187 (3) Å	Mo *K*α radiation, λ = 0.71073 Å
*b* = 11.4119 (3) Å	Cell parameters from 13838 reflections
*c* = 12.1012 (4) Å	θ = 2.7–29.0°
α = 70.437 (3)°	µ = 7.05 mm^−^^1^
β = 73.688 (3)°	*T* = 100 K
γ = 87.038 (2)°	Block, orange
*V* = 1037.76 (6) Å^3^	0.3 × 0.3 × 0.15 mm

### (I) trans-(2-Aminopyridine-κN)dichlorido{4-ethoxycarbonylmethoxy-3-methoxy-1-[(2,3-η)-prop-2-en-1-yl]benzene}platinum(II) Data collection

**Table:**

Agilent SuperNova diffractometer (single source at offset, Eos detector)	4241 independent reflections
Radiation source: micro-focus sealed X-ray tube, SuperNova (Mo) X-ray Source	4006 reflections with *I* > 2σ(*I*)
Mirror monochromator	*R*_int_ = 0.052
Detector resolution: 15.9631 pixels mm^-1^	θ_max_ = 26.4°, θ_min_ = 2.6°
ω scans	*h* = −10→10
Absorption correction: multi-scan (CrysAlis PRO; Rigaku Oxford Diffraction, 2015)	*k* = −14→14
*T*_min_ = 0.406, *T*_max_ = 1.000	*l* = −15→15
21329 measured reflections	

### (I) trans-(2-Aminopyridine-κN)dichlorido{4-ethoxycarbonylmethoxy-3-methoxy-1-[(2,3-η)-prop-2-en-1-yl]benzene}platinum(II) Refinement

**Table:**

Refinement on *F*^2^	26 restraints
Least-squares matrix: full	Hydrogen site location: inferred from neighbouring sites
*R*[*F*^2^ > 2σ(*F*^2^)] = 0.029	H-atom parameters constrained
*wR*(*F*^2^) = 0.065	*w* = 1/[σ^2^(*F*_o_^2^) + (0.0193*P*)^2^ + 4.6851*P*] where *P* = (*F*_o_^2^ + 2*F*_c_^2^)/3
*S* = 1.04	(Δ/σ)_max_ = 0.001
4241 reflections	Δρ_max_ = 1.41 e Å^−^^3^
265 parameters	Δρ_min_ = −2.25 e Å^−^^3^

### (I) trans-(2-Aminopyridine-κN)dichlorido{4-ethoxycarbonylmethoxy-3-methoxy-1-[(2,3-η)-prop-2-en-1-yl]benzene}platinum(II) Special details

**Table:**

Geometry. All esds (except the esd in the dihedral angle between two l.s. planes) are estimated using the full covariance matrix. The cell esds are taken into account individually in the estimation of esds in distances, angles and torsion angles; correlations between esds in cell parameters are only used when they are defined by crystal symmetry. An approximate (isotropic) treatment of cell esds is used for estimating esds involving l.s. planes.

### (I) trans-(2-Aminopyridine-κN)dichlorido{4-ethoxycarbonylmethoxy-3-methoxy-1-[(2,3-η)-prop-2-en-1-yl]benzene}platinum(II) Fractional atomic coordinates and isotropic or equivalent isotropic displacement parameters (Å^2^)

**Table:**

	*x*	*y*	*z*	*U*_iso_*/*U*_eq_	Occ. (<1)
Pt1	0.11518 (2)	0.08277 (2)	0.62392 (2)	0.02167 (7)	
N2	−0.0430 (4)	0.0375 (3)	0.7993 (3)	0.0155 (7)	
C3	−0.0741 (5)	0.1198 (4)	0.8603 (4)	0.0146 (8)	
C4	−0.1797 (5)	0.0846 (4)	0.9812 (4)	0.0170 (8)	
H4	−0.2041	0.1432	1.0232	0.020*	
C5	−0.2465 (5)	−0.0343 (4)	1.0371 (4)	0.0205 (9)	
H5	−0.3167	−0.0591	1.1187	0.025*	
C6	−0.2119 (6)	−0.1198 (4)	0.9745 (4)	0.0208 (9)	
H6	−0.2573	−0.2030	1.0124	0.025*	
C7	−0.1108 (5)	−0.0804 (4)	0.8570 (4)	0.0175 (9)	
H7	−0.0870	−0.1380	0.8139	0.021*	
N8	−0.0004 (4)	0.2351 (3)	0.8045 (3)	0.0174 (7)	
H8A	0.0666	0.2556	0.7296	0.021*	
H8B	−0.0193	0.2898	0.8429	0.021*	
Cl9	−0.0885 (2)	0.20013 (13)	0.55243 (12)	0.0524 (5)	
Cl10	0.30457 (16)	−0.05058 (16)	0.70053 (13)	0.0431 (4)	
C11	0.2551 (6)	0.0923 (4)	0.4411 (4)	0.0277 (11)	
H11A	0.1965	0.1311	0.3822	0.033*	0.614 (14)
H11B	0.2525	0.0041	0.4747	0.033*	0.614 (14)
H11C	0.3564	0.0494	0.4378	0.033*	0.386 (14)
H11D	0.1567	0.0527	0.4423	0.033*	0.386 (14)
C12A	0.3491 (9)	0.1682 (6)	0.4797 (6)	0.023 (2)	0.614 (14)
H12A	0.4508	0.1263	0.5000	0.028*	0.614 (14)
C12B	0.2510 (13)	0.2166 (8)	0.4441 (9)	0.017 (3)	0.386 (14)
H12B	0.1698	0.2651	0.4012	0.020*	0.386 (14)
C13	0.3685 (9)	0.2936 (6)	0.4434 (6)	0.063 (2)	
H13A	0.2955	0.3289	0.3893	0.075*	0.614 (14)
H13B	0.4857	0.3167	0.3923	0.075*	0.614 (14)
H13C	0.3991	0.3586	0.3616	0.075*	0.386 (14)
H13D	0.4693	0.2450	0.4510	0.075*	0.386 (14)
C14	0.3340 (7)	0.3596 (5)	0.5366 (4)	0.0345 (13)	
C15	0.4254 (6)	0.3359 (4)	0.6219 (4)	0.0270 (11)	
H15	0.5109	0.2775	0.6217	0.032*	
C16	0.3922 (5)	0.3972 (4)	0.7070 (4)	0.0175 (9)	
C17	0.2668 (5)	0.4837 (4)	0.7075 (4)	0.0170 (8)	
C18	0.1784 (6)	0.5086 (4)	0.6213 (4)	0.0243 (10)	
H18	0.0940	0.5679	0.6201	0.029*	
C19	0.2133 (7)	0.4467 (5)	0.5364 (4)	0.0315 (12)	
H19	0.1527	0.4649	0.4773	0.038*	
O20	0.4752 (4)	0.3819 (3)	0.7940 (3)	0.0220 (7)	
C21	0.6142 (6)	0.3034 (5)	0.7900 (5)	0.0313 (12)	
H21A	0.6947	0.3334	0.7087	0.047*	
H21B	0.5748	0.2180	0.8068	0.047*	
H21C	0.6687	0.3047	0.8516	0.047*	
O22	0.2431 (4)	0.5390 (3)	0.7957 (3)	0.0178 (6)	
C23	0.1152 (5)	0.6250 (4)	0.8002 (4)	0.0187 (9)	
H23A	0.1245	0.6827	0.7165	0.022*	
H23B	0.1309	0.6750	0.8497	0.022*	
C24	−0.0586 (5)	0.5614 (4)	0.8548 (4)	0.0163 (8)	
O25	−0.0878 (4)	0.4502 (3)	0.9044 (3)	0.0201 (6)	
O26	−0.1749 (3)	0.6460 (3)	0.8437 (3)	0.0170 (6)	
C27	−0.3490 (5)	0.5976 (4)	0.9030 (4)	0.0188 (9)	
H27A	−0.3670	0.5590	0.9923	0.023*	
H27B	−0.3737	0.5333	0.8710	0.023*	
C28	−0.4627 (5)	0.7039 (4)	0.8770 (4)	0.0192 (9)	
H28A	−0.4425	0.7425	0.7885	0.029*	
H28B	−0.4399	0.7657	0.9113	0.029*	
H28C	−0.5798	0.6727	0.9143	0.029*	

### (I) trans-(2-Aminopyridine-κN)dichlorido{4-ethoxycarbonylmethoxy-3-methoxy-1-[(2,3-η)-prop-2-en-1-yl]benzene}platinum(II) Atomic displacement parameters (Å^2^)

**Table:**

	*U*^11^	*U*^22^	*U*^33^	*U*^12^	*U*^13^	*U*^23^
Pt1	0.03045 (11)	0.01466 (10)	0.01438 (10)	−0.00576 (7)	0.00493 (7)	−0.00608 (7)
N2	0.0166 (17)	0.0131 (17)	0.0137 (17)	0.0016 (14)	−0.0029 (14)	−0.0015 (14)
C3	0.0140 (19)	0.013 (2)	0.018 (2)	0.0038 (15)	−0.0069 (16)	−0.0051 (16)
C4	0.016 (2)	0.020 (2)	0.015 (2)	0.0041 (16)	−0.0037 (16)	−0.0067 (17)
C5	0.014 (2)	0.027 (2)	0.017 (2)	−0.0024 (17)	−0.0031 (17)	−0.0031 (18)
C6	0.023 (2)	0.015 (2)	0.020 (2)	−0.0071 (17)	−0.0048 (18)	0.0001 (17)
C7	0.021 (2)	0.015 (2)	0.016 (2)	−0.0006 (17)	−0.0051 (17)	−0.0042 (17)
N8	0.0212 (18)	0.0122 (17)	0.0168 (18)	0.0005 (14)	−0.0011 (14)	−0.0054 (14)
Cl9	0.1016 (13)	0.0365 (8)	0.0210 (6)	0.0429 (8)	−0.0248 (7)	−0.0113 (6)
Cl10	0.0236 (6)	0.0824 (11)	0.0382 (7)	0.0192 (7)	−0.0099 (5)	−0.0404 (8)
C11	0.038 (3)	0.022 (2)	0.014 (2)	−0.007 (2)	0.0094 (19)	−0.0080 (18)
C12A	0.020 (4)	0.024 (4)	0.016 (4)	−0.007 (3)	0.009 (3)	−0.006 (3)
C12B	0.024 (6)	0.020 (5)	0.005 (5)	−0.001 (4)	−0.003 (4)	−0.004 (4)
C13	0.071 (4)	0.072 (4)	0.038 (3)	−0.051 (4)	0.030 (3)	−0.040 (3)
C14	0.040 (3)	0.039 (3)	0.018 (2)	−0.031 (3)	0.015 (2)	−0.015 (2)
C15	0.022 (2)	0.023 (2)	0.028 (3)	−0.0113 (19)	0.0111 (19)	−0.012 (2)
C16	0.016 (2)	0.013 (2)	0.019 (2)	−0.0036 (16)	−0.0001 (17)	−0.0037 (17)
C17	0.015 (2)	0.017 (2)	0.015 (2)	−0.0030 (16)	−0.0003 (16)	−0.0024 (17)
C18	0.024 (2)	0.022 (2)	0.020 (2)	−0.0099 (19)	−0.0062 (19)	0.0024 (19)
C19	0.038 (3)	0.036 (3)	0.014 (2)	−0.023 (2)	−0.004 (2)	0.001 (2)
O20	0.0160 (15)	0.0223 (16)	0.0280 (17)	0.0099 (13)	−0.0059 (13)	−0.0104 (14)
C21	0.020 (2)	0.028 (3)	0.038 (3)	0.012 (2)	−0.001 (2)	−0.007 (2)
O22	0.0159 (14)	0.0168 (15)	0.0246 (16)	0.0060 (12)	−0.0075 (12)	−0.0112 (13)
C23	0.016 (2)	0.012 (2)	0.028 (2)	0.0032 (16)	−0.0051 (18)	−0.0079 (18)
C24	0.018 (2)	0.015 (2)	0.016 (2)	0.0015 (16)	−0.0059 (16)	−0.0042 (17)
O25	0.0216 (16)	0.0144 (15)	0.0222 (16)	−0.0002 (12)	−0.0033 (13)	−0.0057 (13)
O26	0.0130 (14)	0.0128 (14)	0.0220 (16)	0.0001 (11)	−0.0015 (12)	−0.0044 (12)
C27	0.013 (2)	0.019 (2)	0.021 (2)	−0.0030 (17)	0.0005 (17)	−0.0057 (18)
C28	0.015 (2)	0.024 (2)	0.017 (2)	0.0017 (17)	−0.0038 (17)	−0.0062 (18)

### (I) trans-(2-Aminopyridine-κN)dichlorido{4-ethoxycarbonylmethoxy-3-methoxy-1-[(2,3-η)-prop-2-en-1-yl]benzene}platinum(II) Geometric parameters (Å, º)

**Table:**

Pt1—N2	2.066 (3)	C13—H13C	0.9900
Pt1—Cl9	2.2860 (14)	C13—H13D	0.9900
Pt1—Cl10	2.2990 (14)	C13—C14	1.513 (7)
Pt1—C11	2.161 (4)	C14—C15	1.395 (8)
Pt1—C12A	2.221 (7)	C14—C19	1.376 (8)
Pt1—C12B	2.217 (10)	C15—H15	0.9500
N2—C3	1.352 (5)	C15—C16	1.389 (6)
N2—C7	1.358 (5)	C16—C17	1.399 (6)
C3—C4	1.412 (6)	C16—O20	1.375 (5)
C3—N8	1.348 (5)	C17—C18	1.386 (6)
C4—H4	0.9500	C17—O22	1.377 (5)
C4—C5	1.364 (6)	C18—H18	0.9500
C5—H5	0.9500	C18—C19	1.391 (7)
C5—C6	1.397 (6)	C19—H19	0.9500
C6—H6	0.9500	O20—C21	1.426 (5)
C6—C7	1.367 (6)	C21—H21A	0.9800
C7—H7	0.9500	C21—H21B	0.9800
N8—H8A	0.8800	C21—H21C	0.9800
N8—H8B	0.8800	O22—C23	1.413 (5)
C11—H11A	0.9500	C23—H23A	0.9900
C11—H11B	0.9500	C23—H23B	0.9900
C11—H11C	0.9500	C23—C24	1.518 (6)
C11—H11D	0.9500	C24—O25	1.211 (5)
C11—C12A	1.458 (8)	C24—O26	1.333 (5)
C11—C12B	1.429 (9)	O26—C27	1.467 (5)
C12A—H12A	1.0000	C27—H27A	0.9900
C12A—C13	1.353 (9)	C27—H27B	0.9900
C12B—H12B	1.0000	C27—C28	1.502 (6)
C12B—C13	1.344 (9)	C28—H28A	0.9800
C13—H13A	0.9900	C28—H28B	0.9800
C13—H13B	0.9900	C28—H28C	0.9800
			
N2—Pt1—Cl9	89.18 (10)	C12A—C13—H13B	107.2
N2—Pt1—Cl10	88.85 (10)	C12A—C13—C14	120.4 (6)
N2—Pt1—C11	167.04 (16)	C12B—C13—H13C	107.2
N2—Pt1—C12A	153.0 (2)	C12B—C13—H13D	107.2
N2—Pt1—C12B	153.2 (3)	C12B—C13—C14	120.7 (6)
Cl9—Pt1—Cl10	174.90 (6)	H13A—C13—H13B	106.8
C11—Pt1—Cl9	91.10 (15)	H13C—C13—H13D	106.8
C11—Pt1—Cl10	89.77 (15)	C14—C13—H13A	107.2
C11—Pt1—C12A	38.8 (2)	C14—C13—H13B	107.2
C11—Pt1—C12B	38.1 (2)	C14—C13—H13C	107.2
C12A—Pt1—Cl9	103.0 (2)	C14—C13—H13D	107.2
C12A—Pt1—Cl10	80.8 (2)	C15—C14—C13	121.1 (6)
C12B—Pt1—Cl9	75.1 (3)	C19—C14—C13	119.9 (6)
C12B—Pt1—Cl10	108.4 (3)	C19—C14—C15	119.0 (5)
C3—N2—Pt1	122.0 (3)	C14—C15—H15	119.8
C3—N2—C7	119.1 (4)	C16—C15—C14	120.5 (5)
C7—N2—Pt1	118.9 (3)	C16—C15—H15	119.8
N2—C3—C4	120.4 (4)	C15—C16—C17	120.0 (4)
N8—C3—N2	118.8 (4)	O20—C16—C15	125.3 (4)
N8—C3—C4	120.8 (4)	O20—C16—C17	114.6 (4)
C3—C4—H4	120.3	C18—C17—C16	119.3 (4)
C5—C4—C3	119.4 (4)	O22—C17—C16	115.6 (4)
C5—C4—H4	120.3	O22—C17—C18	125.1 (4)
C4—C5—H5	119.9	C17—C18—H18	120.0
C4—C5—C6	120.1 (4)	C17—C18—C19	120.0 (5)
C6—C5—H5	119.9	C19—C18—H18	120.0
C5—C6—H6	120.9	C14—C19—C18	121.2 (5)
C7—C6—C5	118.1 (4)	C14—C19—H19	119.4
C7—C6—H6	120.9	C18—C19—H19	119.4
N2—C7—C6	122.9 (4)	C16—O20—C21	116.7 (4)
N2—C7—H7	118.5	O20—C21—H21A	109.5
C6—C7—H7	118.5	O20—C21—H21B	109.5
C3—N8—H8A	120.0	O20—C21—H21C	109.5
C3—N8—H8B	120.0	H21A—C21—H21B	109.5
H8A—N8—H8B	120.0	H21A—C21—H21C	109.5
Pt1—C11—H11A	112.8	H21B—C21—H21C	109.5
Pt1—C11—H11B	84.7	C17—O22—C23	117.0 (3)
Pt1—C11—H11C	113.4	O22—C23—H23A	109.2
Pt1—C11—H11D	83.9	O22—C23—H23B	109.2
H11A—C11—H11B	120.0	O22—C23—C24	112.2 (3)
H11C—C11—H11D	120.0	H23A—C23—H23B	107.9
C12A—C11—Pt1	72.8 (3)	C24—C23—H23A	109.2
C12A—C11—H11A	120.0	C24—C23—H23B	109.2
C12A—C11—H11B	120.0	O25—C24—C23	125.1 (4)
C12B—C11—Pt1	73.1 (4)	O25—C24—O26	124.8 (4)
C12B—C11—H11C	120.0	O26—C24—C23	110.1 (3)
C12B—C11—H11D	120.0	C24—O26—C27	115.6 (3)
Pt1—C12A—H12A	111.8	O26—C27—H27A	110.0
C11—C12A—Pt1	68.3 (3)	O26—C27—H27B	110.0
C11—C12A—H12A	111.8	O26—C27—C28	108.4 (3)
C13—C12A—Pt1	116.3 (5)	H27A—C27—H27B	108.4
C13—C12A—C11	129.2 (6)	C28—C27—H27A	110.0
C13—C12A—H12A	111.8	C28—C27—H27B	110.0
Pt1—C12B—H12B	110.3	C27—C28—H28A	109.5
C11—C12B—Pt1	68.8 (4)	C27—C28—H28B	109.5
C11—C12B—H12B	110.3	C27—C28—H28C	109.5
C13—C12B—Pt1	116.9 (6)	H28A—C28—H28B	109.5
C13—C12B—C11	132.6 (8)	H28A—C28—H28C	109.5
C13—C12B—H12B	110.3	H28B—C28—H28C	109.5
C12A—C13—H13A	107.2		
			
Pt1—N2—C3—C4	−178.4 (3)	C13—C14—C19—C18	179.8 (4)
Pt1—N2—C3—N8	0.5 (5)	C14—C15—C16—C17	−0.2 (6)
Pt1—N2—C7—C6	177.6 (3)	C14—C15—C16—O20	−179.4 (4)
Pt1—C11—C12A—C13	−106.9 (7)	C15—C14—C19—C18	−1.7 (7)
Pt1—C11—C12B—C13	107.7 (11)	C15—C16—C17—C18	−0.9 (6)
Pt1—C12A—C13—C14	46.1 (8)	C15—C16—C17—O22	179.7 (4)
Pt1—C12B—C13—C14	−44.9 (10)	C15—C16—O20—C21	4.8 (6)
N2—C3—C4—C5	1.8 (6)	C16—C17—C18—C19	0.8 (6)
C3—N2—C7—C6	1.0 (6)	C16—C17—O22—C23	−178.8 (4)
C3—C4—C5—C6	−0.7 (6)	C17—C16—O20—C21	−174.4 (4)
C4—C5—C6—C7	−0.2 (6)	C17—C18—C19—C14	0.5 (7)
C5—C6—C7—N2	0.1 (7)	C17—O22—C23—C24	74.6 (5)
C7—N2—C3—C4	−1.9 (6)	C18—C17—O22—C23	1.9 (6)
C7—N2—C3—N8	177.0 (4)	C19—C14—C15—C16	1.5 (7)
N8—C3—C4—C5	−177.1 (4)	O20—C16—C17—C18	178.4 (4)
C11—C12A—C13—C14	128.7 (7)	O20—C16—C17—O22	−1.0 (5)
C11—C12B—C13—C14	−130.1 (10)	O22—C17—C18—C19	−179.9 (4)
C12A—C13—C14—C15	61.2 (8)	O22—C23—C24—O25	9.5 (6)
C12A—C13—C14—C19	−120.3 (7)	O22—C23—C24—O26	−172.5 (3)
C12B—C13—C14—C15	116.1 (8)	C23—C24—O26—C27	−174.6 (3)
C12B—C13—C14—C19	−65.4 (9)	C24—O26—C27—C28	−177.6 (3)
C13—C14—C15—C16	−180.0 (4)	O25—C24—O26—C27	3.5 (6)

### (I) trans-(2-Aminopyridine-κN)dichlorido{4-ethoxycarbonylmethoxy-3-methoxy-1-[(2,3-η)-prop-2-en-1-yl]benzene}platinum(II) Hydrogen-bond geometry (Å, º)

*Cg*1 is the centroid of the C14–C19 ring.

**Table:**

*D*—H···*A*	*D*—H	H···*A*	*D*···*A*	*D*—H···*A*
N8—H8*B*···O25	0.88	2.19	3.054 (5)	167
C7—H7···O26^i^	0.95	2.49	3.258 (6)	138
C18—H18···Cl9^ii^	0.95	2.78	3.460 (5)	130
N8—H8*A*···*Cg*1	0.88	2.53	3.166 (4)	129

Symmetry codes: (i) *x*, *y*−1, *z*; (ii) −*x*, −*y*+1, −*z*+1.

### (II) (2-Aminopyridine-κN)chlorido{5-ethoxycarbonylmethoxy-4-methoxy-1-[(2,3-η)-prop-2-en-1-yl]phenyl-κC1}platinum(II) Crystal data

**Table:**

[Pt(C_14_H_17_O_4_)Cl(C_5_H_6_N_2_)]	*F*(000) = 1112
*M**_r_* = 573.93	*D*_x_ = 1.984 Mg m^−^^3^
Monoclinic, *P*2_1_/*c*	Mo *K*α radiation, λ = 0.71073 Å
*a* = 15.1198 (5) Å	Cell parameters from 14326 reflections
*b* = 10.0855 (2) Å	θ = 3.2–29.1°
*c* = 14.1855 (4) Å	µ = 7.47 mm^−^^1^
β = 117.329 (4)°	*T* = 100 K
*V* = 1921.72 (11) Å^3^	Block, white
*Z* = 4	0.3 × 0.3 × 0.2 mm

### (II) (2-Aminopyridine-κN)chlorido{5-ethoxycarbonylmethoxy-4-methoxy-1-[(2,3-η)-prop-2-en-1-yl]phenyl-κC1}platinum(II) Data collection

**Table:**

Agilent SuperNova diffractometer (single source at offset, Eos detector)	3928 independent reflections
Radiation source: micro-focus sealed X-ray tube, SuperNova (Mo) X-ray Source	3715 reflections with *I* > 2σ(*I*)
Mirror monochromator	*R*_int_ = 0.033
Detector resolution: 15.9631 pixels mm^-1^	θ_max_ = 26.3°, θ_min_ = 2.5°
ω scans	*h* = −18→18
Absorption correction: multi-scan (CrysAlis PRO; Rigaku Oxford Diffraction, 2015)	*k* = −12→12
*T*_min_ = 0.314, *T*_max_ = 1.000	*l* = −17→17
20268 measured reflections	

### (II) (2-Aminopyridine-κN)chlorido{5-ethoxycarbonylmethoxy-4-methoxy-1-[(2,3-η)-prop-2-en-1-yl]phenyl-κC1}platinum(II) Refinement

**Table:**

Refinement on *F*^2^	0 restraints
Least-squares matrix: full	Hydrogen site location: inferred from neighbouring sites
*R*[*F*^2^ > 2σ(*F*^2^)] = 0.016	H-atom parameters constrained
*wR*(*F*^2^) = 0.038	*w* = 1/[σ^2^(*F*_o_^2^) + (0.0159*P*)^2^ + 1.3858*P*] where *P* = (*F*_o_^2^ + 2*F*_c_^2^)/3
*S* = 1.06	(Δ/σ)_max_ < 0.001
3928 reflections	Δρ_max_ = 0.69 e Å^−^^3^
246 parameters	Δρ_min_ = −1.04 e Å^−^^3^

### (II) (2-Aminopyridine-κN)chlorido{5-ethoxycarbonylmethoxy-4-methoxy-1-[(2,3-η)-prop-2-en-1-yl]phenyl-κC1}platinum(II) Special details

**Table:**

Geometry. All esds (except the esd in the dihedral angle between two l.s. planes) are estimated using the full covariance matrix. The cell esds are taken into account individually in the estimation of esds in distances, angles and torsion angles; correlations between esds in cell parameters are only used when they are defined by crystal symmetry. An approximate (isotropic) treatment of cell esds is used for estimating esds involving l.s. planes.

### (II) (2-Aminopyridine-κN)chlorido{5-ethoxycarbonylmethoxy-4-methoxy-1-[(2,3-η)-prop-2-en-1-yl]phenyl-κC1}platinum(II) Fractional atomic coordinates and isotropic or equivalent isotropic displacement parameters (Å^2^)

**Table:**

	*x*	*y*	*z*	*U*_iso_*/*U*_eq_	
Pt1	0.10189 (2)	0.16233 (2)	0.26543 (2)	0.00950 (4)	
N2	−0.03286 (15)	0.2169 (2)	0.12855 (16)	0.0120 (4)	
C3	−0.10502 (19)	0.1243 (3)	0.0838 (2)	0.0152 (5)	
H3	−0.0934	0.0380	0.1141	0.018*	
C4	−0.1940 (2)	0.1495 (3)	−0.0030 (2)	0.0184 (6)	
H4	−0.2437	0.0828	−0.0320	0.022*	
C5	−0.2097 (2)	0.2756 (3)	−0.0476 (2)	0.0185 (6)	
H5	−0.2705	0.2958	−0.1084	0.022*	
C6	−0.1378 (2)	0.3701 (3)	−0.0039 (2)	0.0172 (6)	
H6	−0.1479	0.4561	−0.0344	0.021*	
C7	−0.0487 (2)	0.3395 (2)	0.0864 (2)	0.0131 (5)	
N8	0.02411 (16)	0.4301 (2)	0.13082 (18)	0.0190 (5)	
H8A	0.0804	0.4087	0.1861	0.023*	
H8B	0.0154	0.5109	0.1046	0.023*	
C9	0.1753 (2)	0.1410 (3)	0.1694 (2)	0.0165 (6)	
H9A	0.2434	0.1627	0.2122	0.020*	
H9B	0.1320	0.2033	0.1191	0.020*	
C10	0.13895 (19)	0.0178 (3)	0.18011 (19)	0.0141 (5)	
H10	0.0818	−0.0176	0.1146	0.017*	
C11	0.20439 (19)	−0.0853 (3)	0.25867 (19)	0.0140 (5)	
H11A	0.1626	−0.1585	0.2626	0.017*	
H11B	0.2511	−0.1227	0.2345	0.017*	
C12	0.26227 (18)	−0.0239 (2)	0.36696 (18)	0.0106 (5)	
C13	0.22571 (18)	0.0934 (2)	0.38748 (19)	0.0105 (5)	
C14	0.27648 (18)	0.1489 (2)	0.48888 (19)	0.0102 (5)	
H14	0.2536	0.2298	0.5043	0.012*	
C15	0.35958 (18)	0.0873 (2)	0.56683 (18)	0.0104 (5)	
C16	0.39390 (17)	−0.0322 (2)	0.54590 (18)	0.0099 (5)	
C17	0.34544 (18)	−0.0865 (2)	0.44517 (19)	0.0105 (5)	
H17	0.3691	−0.1666	0.4295	0.013*	
O18	0.47426 (13)	−0.08947 (17)	0.62958 (13)	0.0129 (4)	
C19	0.52470 (19)	−0.1911 (3)	0.6034 (2)	0.0148 (5)	
H19A	0.5461	−0.1566	0.5526	0.022*	
H19B	0.4797	−0.2664	0.5719	0.022*	
H19C	0.5831	−0.2202	0.6679	0.022*	
O20	0.40683 (13)	0.14275 (17)	0.66777 (13)	0.0113 (4)	
C21	0.50526 (18)	0.1854 (2)	0.6969 (2)	0.0124 (5)	
H21A	0.5375	0.1211	0.6697	0.015*	
H21B	0.5436	0.1867	0.7752	0.015*	
C22	0.50842 (19)	0.3214 (3)	0.65427 (19)	0.0135 (5)	
O23	0.43843 (14)	0.39064 (19)	0.60292 (15)	0.0208 (4)	
O24	0.60368 (13)	0.35149 (17)	0.68185 (14)	0.0140 (4)	
C25	0.6206 (2)	0.4778 (3)	0.6433 (2)	0.0177 (6)	
H25A	0.5806	0.5482	0.6543	0.021*	
H25B	0.6007	0.4719	0.5665	0.021*	
C26	0.7291 (2)	0.5093 (3)	0.7039 (2)	0.0240 (6)	
H26A	0.7436	0.5898	0.6749	0.036*	
H26B	0.7681	0.4353	0.6978	0.036*	
H26C	0.7466	0.5234	0.7788	0.036*	
Cl27	0.04241 (4)	0.25459 (6)	0.37522 (5)	0.01518 (13)	

### (II) (2-Aminopyridine-κN)chlorido{5-ethoxycarbonylmethoxy-4-methoxy-1-[(2,3-η)-prop-2-en-1-yl]phenyl-κC1}platinum(II) Atomic displacement parameters (Å^2^)

**Table:**

	*U*^11^	*U*^22^	*U*^33^	*U*^12^	*U*^13^	*U*^23^
Pt1	0.00860 (6)	0.00919 (6)	0.00852 (6)	0.00084 (3)	0.00203 (4)	0.00005 (3)
N2	0.0111 (10)	0.0127 (11)	0.0102 (10)	0.0025 (9)	0.0033 (8)	0.0001 (8)
C3	0.0147 (13)	0.0158 (13)	0.0155 (13)	−0.0001 (11)	0.0074 (11)	−0.0018 (11)
C4	0.0147 (14)	0.0216 (15)	0.0171 (13)	0.0003 (11)	0.0058 (11)	−0.0074 (11)
C5	0.0140 (13)	0.0283 (16)	0.0101 (12)	0.0096 (12)	0.0029 (10)	−0.0020 (11)
C6	0.0182 (14)	0.0192 (14)	0.0134 (13)	0.0088 (11)	0.0066 (11)	0.0030 (11)
C7	0.0150 (13)	0.0148 (14)	0.0122 (12)	0.0055 (10)	0.0084 (11)	0.0014 (10)
N8	0.0184 (12)	0.0116 (11)	0.0218 (12)	0.0009 (9)	0.0047 (10)	0.0043 (9)
C9	0.0136 (13)	0.0237 (15)	0.0113 (12)	0.0045 (11)	0.0051 (11)	0.0000 (11)
C10	0.0145 (13)	0.0167 (14)	0.0093 (11)	0.0025 (11)	0.0039 (10)	−0.0050 (10)
C11	0.0127 (13)	0.0128 (13)	0.0129 (12)	0.0019 (10)	0.0028 (10)	−0.0034 (10)
C12	0.0103 (12)	0.0133 (13)	0.0088 (11)	−0.0013 (10)	0.0049 (10)	−0.0018 (10)
C13	0.0086 (12)	0.0111 (13)	0.0125 (12)	0.0000 (10)	0.0054 (10)	0.0015 (10)
C14	0.0106 (12)	0.0090 (12)	0.0122 (12)	0.0005 (9)	0.0063 (10)	−0.0004 (9)
C15	0.0109 (12)	0.0128 (13)	0.0088 (11)	−0.0051 (10)	0.0056 (10)	−0.0019 (9)
C16	0.0080 (11)	0.0117 (13)	0.0093 (11)	0.0004 (10)	0.0034 (9)	0.0033 (9)
C17	0.0123 (12)	0.0083 (12)	0.0127 (12)	0.0015 (10)	0.0072 (10)	0.0009 (10)
O18	0.0132 (9)	0.0119 (9)	0.0107 (8)	0.0044 (7)	0.0031 (7)	0.0026 (7)
C19	0.0134 (13)	0.0159 (13)	0.0154 (13)	0.0049 (11)	0.0069 (11)	0.0043 (11)
O20	0.0100 (9)	0.0159 (9)	0.0079 (8)	−0.0030 (7)	0.0039 (7)	−0.0033 (7)
C21	0.0103 (12)	0.0146 (13)	0.0103 (12)	−0.0017 (10)	0.0031 (10)	−0.0010 (10)
C22	0.0142 (13)	0.0156 (14)	0.0088 (12)	−0.0022 (10)	0.0036 (10)	−0.0051 (10)
O23	0.0140 (10)	0.0204 (10)	0.0204 (10)	0.0014 (8)	0.0014 (8)	0.0044 (8)
O24	0.0126 (9)	0.0120 (9)	0.0178 (9)	−0.0010 (7)	0.0072 (8)	0.0013 (7)
C25	0.0212 (14)	0.0124 (14)	0.0179 (13)	−0.0023 (11)	0.0076 (11)	0.0017 (11)
C26	0.0211 (15)	0.0194 (15)	0.0300 (16)	−0.0059 (12)	0.0103 (13)	0.0030 (12)
Cl27	0.0151 (3)	0.0148 (3)	0.0144 (3)	0.0038 (2)	0.0058 (2)	−0.0020 (2)

### (II) (2-Aminopyridine-κN)chlorido{5-ethoxycarbonylmethoxy-4-methoxy-1-[(2,3-η)-prop-2-en-1-yl]phenyl-κC1}platinum(II) Geometric parameters (Å, º)

**Table:**

Pt1—N2	2.143 (2)	C12—C17	1.389 (3)
Pt1—C9	2.127 (3)	C13—C14	1.399 (3)
Pt1—C10	2.127 (2)	C14—H14	0.9500
Pt1—C13	2.003 (2)	C14—C15	1.382 (3)
Pt1—Cl27	2.3209 (6)	C15—C16	1.397 (4)
N2—C3	1.353 (3)	C15—O20	1.391 (3)
N2—C7	1.346 (3)	C16—C17	1.385 (3)
C3—H3	0.9500	C16—O18	1.376 (3)
C3—C4	1.367 (4)	C17—H17	0.9500
C4—H4	0.9500	O18—C19	1.425 (3)
C4—C5	1.392 (4)	C19—H19A	0.9800
C5—H5	0.9500	C19—H19B	0.9800
C5—C6	1.363 (4)	C19—H19C	0.9800
C6—H6	0.9500	O20—C21	1.416 (3)
C6—C7	1.402 (4)	C21—H21A	0.9900
C7—N8	1.345 (3)	C21—H21B	0.9900
N8—H8A	0.8800	C21—C22	1.509 (4)
N8—H8B	0.8800	C22—O23	1.196 (3)
C9—H9A	0.9500	C22—O24	1.343 (3)
C9—H9B	0.9500	O24—C25	1.454 (3)
C9—C10	1.395 (4)	C25—H25A	0.9900
C10—H10	1.0000	C25—H25B	0.9900
C10—C11	1.514 (3)	C25—C26	1.495 (4)
C11—H11A	0.9900	C26—H26A	0.9800
C11—H11B	0.9900	C26—H26B	0.9800
C11—C12	1.509 (3)	C26—H26C	0.9800
C12—C13	1.391 (4)		
			
N2—Pt1—Cl27	90.28 (6)	C13—C12—C11	117.5 (2)
C9—Pt1—N2	90.24 (9)	C17—C12—C11	121.0 (2)
C9—Pt1—C10	38.29 (10)	C17—C12—C13	121.3 (2)
C9—Pt1—Cl27	161.33 (7)	C12—C13—Pt1	114.69 (17)
C10—Pt1—N2	92.85 (9)	C12—C13—C14	118.2 (2)
C10—Pt1—Cl27	160.20 (7)	C14—C13—Pt1	127.13 (19)
C13—Pt1—N2	174.40 (9)	C13—C14—H14	119.6
C13—Pt1—C9	87.89 (10)	C15—C14—C13	120.7 (2)
C13—Pt1—C10	82.42 (10)	C15—C14—H14	119.6
C13—Pt1—Cl27	93.18 (7)	C14—C15—C16	120.5 (2)
C3—N2—Pt1	118.29 (17)	C14—C15—O20	119.5 (2)
C7—N2—Pt1	122.69 (17)	O20—C15—C16	120.0 (2)
C7—N2—C3	119.0 (2)	C17—C16—C15	119.2 (2)
N2—C3—H3	118.6	O18—C16—C15	116.5 (2)
N2—C3—C4	122.8 (3)	O18—C16—C17	124.3 (2)
C4—C3—H3	118.6	C12—C17—H17	120.0
C3—C4—H4	120.9	C16—C17—C12	120.0 (2)
C3—C4—C5	118.1 (3)	C16—C17—H17	120.0
C5—C4—H4	120.9	C16—O18—C19	116.32 (19)
C4—C5—H5	120.1	O18—C19—H19A	109.5
C6—C5—C4	119.9 (2)	O18—C19—H19B	109.5
C6—C5—H5	120.1	O18—C19—H19C	109.5
C5—C6—H6	120.2	H19A—C19—H19B	109.5
C5—C6—C7	119.6 (3)	H19A—C19—H19C	109.5
C7—C6—H6	120.2	H19B—C19—H19C	109.5
N2—C7—C6	120.6 (2)	C15—O20—C21	113.37 (19)
N8—C7—N2	118.4 (2)	O20—C21—H21A	109.1
N8—C7—C6	121.0 (2)	O20—C21—H21B	109.1
C7—N8—H8A	120.0	O20—C21—C22	112.4 (2)
C7—N8—H8B	120.0	H21A—C21—H21B	107.9
H8A—N8—H8B	120.0	C22—C21—H21A	109.1
Pt1—C9—H9A	107.3	C22—C21—H21B	109.1
Pt1—C9—H9B	91.7	O23—C22—C21	126.4 (2)
H9A—C9—H9B	120.0	O23—C22—O24	125.2 (2)
C10—C9—Pt1	70.86 (15)	O24—C22—C21	108.4 (2)
C10—C9—H9A	120.0	C22—O24—C25	116.0 (2)
C10—C9—H9B	120.0	O24—C25—H25A	110.1
Pt1—C10—H10	115.6	O24—C25—H25B	110.1
C9—C10—Pt1	70.85 (15)	O24—C25—C26	107.8 (2)
C9—C10—H10	115.6	H25A—C25—H25B	108.5
C9—C10—C11	122.4 (2)	C26—C25—H25A	110.1
C11—C10—Pt1	107.81 (16)	C26—C25—H25B	110.1
C11—C10—H10	115.6	C25—C26—H26A	109.5
C10—C11—H11A	109.6	C25—C26—H26B	109.5
C10—C11—H11B	109.6	C25—C26—H26C	109.5
H11A—C11—H11B	108.1	H26A—C26—H26B	109.5
C12—C11—C10	110.2 (2)	H26A—C26—H26C	109.5
C12—C11—H11A	109.6	H26B—C26—H26C	109.5
C12—C11—H11B	109.6		
			
Pt1—N2—C3—C4	179.4 (2)	C13—C12—C17—C16	−0.2 (4)
Pt1—N2—C7—C6	179.41 (18)	C13—C14—C15—C16	−0.5 (4)
Pt1—N2—C7—N8	1.5 (3)	C13—C14—C15—O20	−178.1 (2)
Pt1—C9—C10—C11	−99.4 (2)	C14—C15—C16—C17	1.9 (4)
Pt1—C10—C11—C12	−28.5 (2)	C14—C15—C16—O18	−177.0 (2)
Pt1—C13—C14—C15	177.68 (18)	C14—C15—O20—C21	−115.9 (2)
N2—C3—C4—C5	1.0 (4)	C15—C16—C17—C12	−1.5 (4)
C3—N2—C7—C6	−1.3 (4)	C15—C16—O18—C19	−164.7 (2)
C3—N2—C7—N8	−179.2 (2)	C15—O20—C21—C22	83.1 (3)
C3—C4—C5—C6	−0.7 (4)	C16—C15—O20—C21	66.4 (3)
C4—C5—C6—C7	−0.5 (4)	C17—C12—C13—Pt1	−177.47 (19)
C5—C6—C7—N2	1.5 (4)	C17—C12—C13—C14	1.5 (4)
C5—C6—C7—N8	179.4 (3)	C17—C16—O18—C19	16.5 (3)
C7—N2—C3—C4	0.0 (4)	O18—C16—C17—C12	177.3 (2)
C9—C10—C11—C12	49.7 (3)	O20—C15—C16—C17	179.5 (2)
C10—C11—C12—C13	21.0 (3)	O20—C15—C16—O18	0.6 (3)
C10—C11—C12—C17	−162.9 (2)	O20—C21—C22—O23	1.2 (4)
C11—C12—C13—Pt1	−1.4 (3)	O20—C21—C22—O24	−177.57 (19)
C11—C12—C13—C14	177.6 (2)	C21—C22—O24—C25	177.5 (2)
C11—C12—C17—C16	−176.2 (2)	C22—O24—C25—C26	165.5 (2)
C12—C13—C14—C15	−1.2 (4)	O23—C22—O24—C25	−1.3 (4)

### (II) (2-Aminopyridine-κN)chlorido{5-ethoxycarbonylmethoxy-4-methoxy-1-[(2,3-η)-prop-2-en-1-yl]phenyl-κC1}platinum(II) Hydrogen-bond geometry (Å, º)

*Cg*2 is the centroid of the C12-C17 ring.

**Table:**

*D*—H···*A*	*D*—H	H···*A*	*D*···*A*	*D*—H···*A*
C17—H17···O24^i^	0.95	2.60	3.501 (3)	159
C25—H25*B*···O23^ii^	0.99	2.60	3.449 (3)	144
N8—H8*B*···Cl27^iii^	0.88	2.67	3.413 (2)	143
C19—H19*A*···*Cg*2^i^	0.98	2.63	3.476 (3)	145

Symmetry codes: (i) −*x*+1, −*y*, −*z*+1; (ii) −*x*+1, −*y*+1, −*z*+1; (iii) −*x*, *y*+1/2, −*z*+1/2.
